# Four Proposals to Help Improve the Medical Research Literature

**DOI:** 10.1371/journal.pmed.1001864

**Published:** 2015-09-22

**Authors:** David Moher, Douglas G. Altman

**Affiliations:** 1 Clinical Epidemiology Program, Ottawa Hospital Research Institute; School of Epidemiology, Public Health and Preventive Medicine, Faculty of Medicine, University of Ottawa, Ottawa, Ontario, Canada; 2 Centre for Statistics in Medicine, Nuffield Department of Orthopaedics, Rheumatology and Musculoskeletal Sciences, University of Oxford, Oxford, United Kingdom

## Abstract

David Moher and Douglas Altman outline four potential interventions that may improve the quality of peer-reviewed medical research publications.

**Summary Points**

- The evidence base underpinning clinical practice is deeply flawed.
- There must be better value gained from resources invested in medical research.
- We make four proposals: (1) introducing publications officers; (2) developing core competencies for editors and peer reviewers, around which (3) training can be tailored; and (4) training authors to write articles fit for purpose.
- All of these ideas need to be piloted and evaluated, and implemented if proven effective.
- We suggest dedicated funding for initiatives aimed at understanding and improving the way that research is conducted and published.
- Academic institutions, funders, publishers, and others should support and implement effective processes to improve the reliability of the medical research literature.

Biomedical journals have existed for hundreds of years. They are still the most important conduit for researchers to report the methods and results of their research. However, published articles are fraught with problems [1–11]. For nearly a century, concerns have been raised about the prevalence of methodological errors in medical research, especially in relation to statistics [1–4]. More recently, numerous reviews have identified additional widespread deficiencies in the reporting of research. Crucial aspects of study methods and results are frequently missing [5–11]. Transparency and reproducibility of research are essential [12,13]. Making a major impact on the quality of reporting and mitigating deficiencies is a huge challenge because no one group has prime responsibility and no single action is likely to have a large impact.

Both of us have been involved in medical research for many years as researchers, authors, peer reviewers, editors, and academics. Here, we discuss four potential contributory actions by journals and educational institutions to help to increase the value of research articles: publications officers, core competency training of medical editors, training authors to write articles “fit for purpose” (in other words, the report is a complete and transparent account of what the researchers did and found, thus maximizing the potential usefulness of the article to a broad range of readers), and training peer reviewers. All four ideas need to be piloted and evaluated, and if proven effective, considered for implementation. For ease of presentation and discussion, these ideas are presented separately. However, we recognize they are intertwined, just as with our own careers. We end the essay with a discussion of possible ways to fund these initiatives.

## Introducing Publications Officers

Universities invest resources at the front end of knowledge generation. Many academic centres employ professionals to help their researchers understand the process of successfully competing for a dizzying number and different types of research applications. In Canada, for example, these people are often known as grants officers or technology transfer officers. Their primary objective is to provide direction, guidance, and timely information to the institute’s scientists relating to grant submissions. After the research monies are secured and the research is conducted, the findings are ready for dissemination through presentations at scientific conferences and journal publications. Unfortunately, this dissemination model is not fully effective. For example, too much research is never published [14,15]. And many research reports that are published display important weaknesses [16].

To help rectify this situation, we propose the introduction of publications officers, who would support and educate researchers, staff, and trainees in universities and research organisations. Their roles and responsibilities could include providing guidance on preparing manuscripts for submission to journals (including adherence to relevant reporting guidelines and the submission procedures); developing seminars on how to write to get published—that is, writing articles that are fit for purpose [17]; harnessing existing resources relevant to manuscript preparation and publication, addressing research integrity and publication ethics; and facilitating internal peer review of manuscripts before submission to journals. Other activities might include facilitating in-depth training on using reporting guidelines when preparing manuscript submissions, regular seminars on issues about publication ethics and research integrity and responsibility, explaining open access options, and providing seminars to the local community on “making sense of science,” such as how authors use “spin” to interpret the results of their research [18].

Some organizations, for example, the International Society for Medical Publication Professionals (http://www.ismpp.org/), provide resources for professional medical writers. They also offer certification (Certified Medical Publication Professional). But there seem to be few comparable opportunities for academic researchers.

Currently there is an inequity in academic institutional thinking—great interest in maximizing the chances of succeeding in grant applications, yet little attention given to maximizing success when the research project has been completed, namely, dissemination, although universities typically employ press officers (communications officers) to maximise opportunities of the media reporting on their scientists’ research. Publications are the tangible output from all of the research activity, so they surely merit serious investment to ensure complete and transparent reports. We believe that introducing publications officers within academic institutions would help to reduce nonpublication and selective publication of research findings, improve the clarity and transparency of the institution’s research output, and help raise the quality and value of their researchers’ publications.

There is, as yet, little experience regarding the ideal publications officer. Backgrounds in education, clinical epidemiology, medical writing, research ethics, or a combination of these would seem appropriate. One of our institutions (Ottawa Hospital Research Institute) very recently employed a publications officer with a background in biology and psychology. As part of this early pilot endeavour, the institution is conducting an evaluation of the effectiveness of the position.

## Core Competencies

### Editors

Scientific editors (and ultimately, editors-in-chief) are accountable for all published material in their journals. Readers should expect them to have processes in place to ensure the quality of the papers they publish and to strive constantly to improve their journals. While well-resourced medical journals [19] have full-time, paid, professional scientific editors, and their publishers may have resources to provide some formal training for their position, the majority of medical journal editors work on a voluntary basis. Such “pro bono” activities are useful if the scientific activities associated with being an editor are of the highest possible standards. Unfortunately, many medical editors who oversee their journals are largely untrained and certainly uncertified. We think this is not the optimal way to instil confidence in readers, provide value for money to funders, or ensure the public can trust the research record.

Some organizations, for example, the World Association of Medical Editors (WAME), provide resources for editors. There are some good websites, such as Committee on Publication Ethics (COPE; http://publicationethics.org/), and blogs, such as Journalology (http://journalology.blogspot.ca/), that provide important information for editors. There are also several short courses on being an editor offered by commercial groups (http://www.pspconsulting.org/medical-short.shtml) and a few large, well-resourced journals offer in-house training for editors (e.g., *The BMJ*).

For a substantial editor training program to work optimally, it must be based primarily on what the broad medical editor community considers to be core competencies for all editors. Other stakeholders need to contribute to this effort, such as publishers, peer reviewers, and authors (researchers). We are unaware, however, of any body of literature identifying what these core competencies are [20]. Given one recent recommendation to use reporting guidelines [16], a core competency might be for editors to have a more thorough knowledge of them, including how best to endorse and implement them and facilitate their use by peer reviewers [20]. One of us is leading the development of a core competency for editors program, one result of which will be a minimum set of evidence-based core competencies. The program has several elements similar to how some reporting guidelines have been successfully developed [21,22].

### Authors

Any efforts aimed at training authors might be considered too late in the knowledge generation cycle. The research has been completed and all that can be done, realistically, is to try to ensure that authors transparently and completely tell readers what they did (methods) and found (results). Recent examples of using guidelines earlier, during the design and conduct of research, include SPIRIT [23] for preparing protocols of randomized trials and PRISMA-P for preparing protocols of systematic reviews [22]. Also, some groups are focused on helping researchers to improve the design and conduct of clinical trials, such as the Core Outcome Measures in Effectiveness Trials (COMET) [24] and TrialForge [25].

Even if such initiatives succeed in improving trial methods, training researchers to write high-quality articles will remain an important and relevant goal. As authors, many researchers have difficulty reporting what they did and found, completely and transparently. Authors need to write papers that are fit for purpose [17]. They need to ensure that every report that includes their name is a completely reported and transparent account of what was done and found, to enable interested readers to replicate the methods and use the results [13]. Collectively, authors are currently not doing a good job reporting their research [16]. If the introduction of publications officers, described above, proves successful, this is one way institutions can help ensure that the manuscripts submitted for publication consideration are the highest quality. This might help reduce waste and increase journal efficiencies, including the peer review process.

For many researchers, writing is difficult. It is an acquired skill that often starts during graduate studies, at which time it should be formally taught and discussed. Developing good writing skills early on can reap benefits throughout a researcher’s career. Formal training in writing, use of reporting guidelines, and issues related to authorship (e.g., attributing authorship, authorship order, author responsibilities) should be mandatory [26]. It is unfortunate that most universities do not promote this type of formal training, given the evidence of the tarnished published literature [1–11,16].

## Training Peer Reviewers

Evidence of the effectiveness of peer review of medical research articles is minimal at best [27]. A recent study found that peer reviewers identified only a fifth of the reporting deficiencies in a cohort of randomised trials [28]. Hundreds of reviews of published articles have shown that peer reviewers fail to detect widespread methodological errors and reporting deficiencies [5–11]. Such dismal results have led some to argue that we should abandon peer review completely [29].

Teaching peer review is probably one of the most important ways to increase trust and confidence in the published research record. There are some commercial short courses on peer review, but to the best of our knowledge there is minimal formal teaching of peer review in academic institutions, the very places where a large amount of research originates. This is also where the next generation of researchers are being trained and cultivated. Furthermore, most journals fail to provide guidance to reviewers on what they expect from them [30]. There are several motivations for completing peer review, including requests from a supervisor, wanting to keep abreast of the latest developments in a specific content area, and altruism. Regardless of the reason for doing peer review, most reviewers do it without training or reward. Most people who perform peer review learn by trial and error and perhaps some mentorship, as we did.

There has been a recent focus on the need to provide comprehensive peer reviewer training and view it more professionally [31]. We believe that to make peer review more effective requires the development of a set of core competencies, after which training programs can be developed. An approach similar to that described for developing core competencies for editors, described above, deserves serious consideration. Such training could be integrated into a broad training curriculum within universities. The objective of formal training is to provide students with the skill set needed to detect manuscripts that are not fit for purpose and help authors to improve them. These skills learned as a peer reviewer can also be used when the peer reviewers write complete and transparent articles as authors—how their study was conducted and what they found. Additionally, such courses need to teach about the role and responsibilities of peer reviewers.

Academic institutions need to take peer review seriously and develop full- or half-semester courses that can be used by students towards their degree. Sufficient institutional resources need to be set aside to ensure these courses can be appropriately developed. These courses should be mandatory for all new graduate students and young researchers.

## Funding

One estimate is that US$240 billion is spent globally, every year, on health research [32]. The outputs from this research are documented in about 3 million articles, of which about half are published by 6,000 publishers in 25,000 journals (with a much larger number of editors). We have briefly described some of the serious problems associated with the published record. In many cases, the information reported cannot be used; in many more, the reporting is biased; and much research is never published at all. It is estimated that 85% [32] of the global investment is avoidable waste (i.e., it is modifiable). This is a bad return on such a large fiscal investment, particularly when there are substantial public monies involved. The status quo is unacceptable, yet there are few signs that major bodies (funders, universities, professional bodies) recognise the need for change.

Just as the problems we have discussed here are large, complex, and not the sole responsibility of any single group, no single stakeholder can or should fund “journalology” (research on research) investigations. That said, funding agencies and others, particularly publishers, are likely central in helping to promote and support the development of sustained programs of investigation in journalology. Perhaps it is possible to start with leadership and support from heads of major funding organizations and large publishers heavily invested in publishing biomedical research. Once such an initial commitment is secured, other relevant groups may join and support the collective effort. It is unclear how best to leverage academic institutions to join this funding effort. One possibility is for them to commit in-kind resources for faculty to have time to develop and pilot these and other similar initiatives.

A very small fraction of funders’ and publishers’ expenditures (say 0.1%) could be set aside for initiatives to reduce waste and improve the quality, and thus value, of research publications, including journalology investigations; it is a legitimate and arguably essential research endeavour. Some small percent of these monies could be used to fund the certification and continuing education training for editors, as well as training for authors and peer reviewers. Some of the investment could be targeted to reach attainable increases in research value, annually, over the next decade. The precise increase could be agreed upon by key players.

In conclusion, publishing medical research is complex; the biomedical research community is failing at it intellectually, fiscally, morally, and ethically [33]. The present state of research publication is unacceptable. We have made four proposals here to help improve the situation, complementing other proposals [19,34]. For these initiatives to succeed, there must be a fundamental shift in how our academic institutions, funding bodies, journals, and publishers perceive the importance of publication practices. Working together, these organizations can help test and potentially implement our proposals and indicate the importance of these and other similar efforts. Ensuring high-quality research publications must become a core activity within their portfolios. The medical publication business needs to be taken much more seriously. Everybody deserves a guarantee of reliable evidence resulting from our research endeavours.
